# Fabrication and Preliminary In Vitro Evaluation of 3D-Printed Alginate Films with Cannabidiol (CBD) and Cannabigerol (CBG) Nanoparticles for Potential Wound-Healing Applications

**DOI:** 10.3390/pharmaceutics14081637

**Published:** 2022-08-05

**Authors:** Paraskevi Kyriaki Monou, Anastasia Maria Mamaligka, Emmanuil K. Tzimtzimis, Dimitrios Tzetzis, Souzan Vergkizi-Nikolakaki, Ioannis S. Vizirianakis, Eleftherios G. Andriotis, Georgios K. Eleftheriadis, Dimitrios G. Fatouros

**Affiliations:** 1 Laboratory of Pharmaceutical Technology, Department of Pharmacy, Aristotle University of Thessaloniki, 54124 Thessaloniki, Greece; 2 Digital Manufacturing and Materials Characterization Laboratory, School of Science and Technology, International Hellenic University, 57001 Thermi, Greece; 3 Department of Microbiology, School of Medicine, Faculty of Health Sciences, Aristotle University of Thessaloniki, 54124 Thessaloniki, Greece; 4 Laboratory of Pharmacology, School of Pharmacy, Faculty of Health Sciences, Aristotle University of Thessaloniki, 54124 Thessaloniki, Greece; 5 Department of Life and Health Sciences, University of Nicosia, Nicosia 2411, Cyprus

**Keywords:** cannabidiol, cannabigerol, cannabinoids, wound-healing, 3D-printing, sodium alginate, Pluronic-F127

## Abstract

In this study, drug carrier nanoparticles comprised of Pluronic-F127 and cannabidiol (CBD) or cannabigerol (CBG) were developed, and their wound healing action was studied. They were further incorporated in 3D printed films based on sodium alginate. The prepared films were characterized morphologically and physicochemically and used to evaluate the drug release profiles of the nanoparticles. Additional studies on their water loss rate, water retention capacity, and 3D-printing shape fidelity were performed. Nanoparticles were characterized physicochemically and for their drug loading performance. They were further assessed for their cytotoxicity (MTT Assay) and wound healing action (Cell Scratch Assay). The in vitro wound-healing study showed that the nanoparticles successfully enhanced wound healing in the first 6 h of application, but in the following 6 h they had an adverse effect. MTT assay studies revealed that in the first 24 h, a concentration of 0.1 mg/mL nanoparticles resulted in satisfactory cell viability, whereas CBG nanoparticles were safe even at 48 h. However, in higher concentrations and after a threshold of 24 h, the cell viability was significantly decreased. The results also presented mono-disperse nano-sized particles with diameters smaller than 200 nm with excellent release profiles and enhanced thermal stability. Their entrapment efficiency and drug loading properties were higher than 97%. The release profiles of the active pharmaceutical ingredients from the films revealed a complete release within 24 h. The fabricated 3D-printed films hold promise for wound healing applications; however, more studies are needed to further elucidate their mechanism of action.

## 1. Introduction

Management of acute and chronic wounds remains a major health challenge and a significant threat to public health and the economy [1]. Wound healing is a dynamic multicellular process governed by complex molecular and biological events. It comprises four temporarily overlapping phases: hemostasis, inflammation, proliferation, and remodeling [2]. The corruption of that sequence can lead to impaired healing or even chronicity of the wound. In addition, speeding up the healing process of acute wounds during surgery could contribute to reduced scar formation. A variety of wound dressings are available or are currently being developed to accelerate the healing process. They serve various purposes, such as protecting the damaged tissue from infection and contamination, providing moisture, absorbing wound exudates, and enhancing cell differentiation and proliferation [3]. Although there are several commercially available types of dressings for different wound types, many drawbacks currently exist, related to inefficient exudate absorption, limited protection against infections, allergic effects, and ineffectualness in providing a stable moist environment [4]. For instance, traditional dressings like gauzes absorb wound moisture and lead to wound surface dehydration, thus decreasing the healing rate. Common approaches employ different polymers such as alginate [2], chitosan [5], pectin [6], polyvinyl alcohol [7] etc. Currently, the successful incorporation of antimicrobial and bioactive molecules in wound dressings is one of the most discussed topics in the field of wound management. Innovative wound dressings have been developed using various processes, such as supercritical impregnation with carbon dioxide [8,9], electrospinning [10], and 3D printing [11].

Cannabis Sativa L. is one of the oldest medicinal plants and its products contain a large variety of chemical compounds and have a long history of uses for medical purposes or as intoxicants. Recent studies reveal that over 1200 different compounds are present in cannabis. More than 100 of them are cannabinoids, a group of C21 or C22 compounds [12,13]. In the last two decades, interest in cannabinoids for therapeutic applications has surged, and knowledge about the therapeutic potential of cannabis products has greatly improved by a large number of those undergoing clinical trials [14]. Despite the resultant legislated legalization in some countries and the spike in publications that began in 2013, research on this topic is still premature and more studies should be conducted to compose a more detailed scientific evidence base on the effects of cannabinoids on the human body.

**Figure 1:**
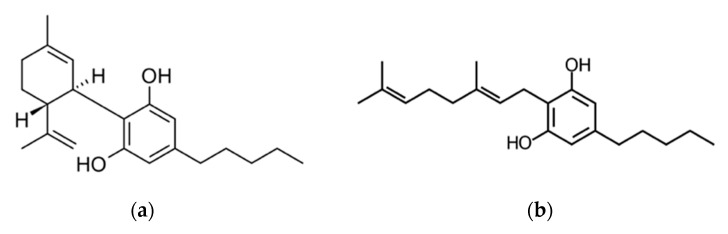
Chemical structures of (**a**) cannabidiol and (**b**) cannabigerol.

The studied cannabinoid is 9-Δ-9-tetrahydrocannabinol (THC-9), mainly due to its psychoactive properties. However, non-psychoactive phytocannabinoids, such as cannabidiol (CBD) and cannabigerol (CBG), have also received noteworthy attention from the scientific community (Figure 1). CBD is one of the most intensively researched cannabinoids owing to its positive therapeutic effects on several medical conditions, i.e., epilepsy [15], schizophrenia [16], anxiety [17], and sepsis [18]. It is also used for the treatment of several skin conditions, including psoriasis, atopic dermatitis, skin cancer, and hair growth disorders [19,20,21]. CBD has also demonstrated anti-inflammatory [22,23], anti-oxidative and wound healing effects [24,25,26,27]. CBG acts as the precursor molecule for the most abundant phytocannabinoids. However, compared to CBD, limited research has been conducted on its pharmacological action. The available studies reveal considerable antibacterial, antioxidant, neuroprotective, and anti-inflammatory activity [28] as well as potential in the treatment of neurological disorders and glaucoma [29]. In 2018, the Food and Drug Administration (FDA) approved Epidiolex^®^, a CBD-containing oral solution for the treatment of seizures associated with two rare and severe forms of epilepsy (Lennox-Gastaut and Dravet syndromes) for patients more than two years old. This was the first FDA-approved drug that contained a purified drug substance derived from cannabis [30]. As scientific research on this topic extends, more drugs based on cannabinoids are expected to get approval in the following years.

Cannabinoid properties are expressed by various mechanisms, including the interactions with the endocannabinoid system (eCS) and other receptors, providing a promising alternative to traditional treatments. The newly found eCS regulates many homeostatic processes, including those involved in wound healing [25].

However, the high hydrophobicity, poor aqueous solubility, and restricted skin permeation, in addition to the thermal and photo-sensitivity of cannabinoids, hinder their usage for topical delivery [31]. Therefore, the development of water-soluble cannabinoid forms is of great importance. Nanonization of cannabinoids by incorporation in a polymeric carrier and formation of a solid suspension could overcome this barrier and enable extended topical delivery [32,33].

Among the different approaches that have been applied to improve the aqueous solubility of lipophilic drugs, the use of amphiphilic polymers that can form micelle-like structures is of great interest. They present a very attractive drug delivery option by providing improved thermodynamic and kinetic stability. Among those, poloxamers have been used in several studies as an efficient drug delivery system [34,35]. One of those is Pluronic-F127 (PF127), a block copolymer that consists of two hydrophilic ethylene oxide (EO) blocks and one hydrophobic propylene oxide (PO) block arranged in an EOx–POy–EOx structure [34]. The PO unimers could interact with the hydrophobic cannabinoids and the EO unimers with the water. The incorporation of lipophilic drugs into the hydrophobic core of the structure can increase their aqueous solubility and stability. The main purpose of drug development is to increase the efficacy of a product and limit its adverse effects. This goal could potentially be achieved by the application of 3D printing in pharmaceutical production. It offers numerous advantages, such as the production of small batches with patient-tailored characteristics, customized implants and prostheses, complex structures for on-demand drug release profiles, and personalized drug dosage forms [36,37]. Accordingly, 3D printers could be installed in pharmacies, hospitals, and remote locations, enabling the on-demand production of customizable dosage forms based on patients’ needs [38,39].

Therefore, the present study is focused on the development of cannabinoid-PF127 nanoparticles, the evaluation of their wound-healing action, and the fabrication of bioactive wound dressings using the extrusion-based 3D-printing technology. There is an emerging trend in using cannabinoids for medical reasons [14]. CBD and CBG have been widely explored regarding their pharmaceutical properties; thus, it is important to develop novel formulations with these actives. 3D printing is a very promising process for producing novel formulations with customizable dosages, suitable for each patient’s needs and fully personalized. This process can also manufacture films with localized actives using a variety of materials [6,40,41]. The 3D-printable inks are comprised of Sodium Alginate (SA), calcium chloride, and a cannabinoid suspension. Two techniques were tested and optimized for the development of nanoparticles. Additional details on the experimental process and the results are described in the following sections. The scientific interest in cannabinoids has skyrocketed in the last few years [14]. However, to the best of our knowledge, there are no articles up to date discussing the wound healing activity of CBG, while for CBD, the related studies are scarce. Still, this is an early-stage study to compare with any other wound healing research.

## 2. Materials and Methods

### 2.1. Materials

CBD (crystals, >99% CBD, 1% terpenes by GC-MS, Enecta, Italy) and CBG (crystals, >99% CBG, 1% terpenes by GC-MS, Enecta, Italy) were kindly donated from Hempoil^®^, Athens, Greece. PF127, ethanol. SA and calcium chloride were supplied from Sigma Aldrich^®^ (Merck KGaA, Darmstadt, Germany). Human adult low-calcium cell lines (HaCaT), Dulbecco’s Modified Eagle Medium (DMEM), Glutamax, Fetal Bovine Serum (FBS), 1% of 10 mg/mL Streptomycin, and 10.000 U/mL Penicillin, trypsin and trypan blue were purchased from Sigma Aldrich (St. Louis, MO, USA). All reagents were of standard analytical grade.

### 2.2. Preparation of CBD and CBG Solid Suspensions

For the preparation of cannabinoid nanoparticle suspensions, two different methods were tested. In method A, accurate amounts of CBD or CBG (5 mg) and PF127 (5 mg) were weighed and solubilized in 99% ethanol (50 μL) while being vortexed. The ethanolic solution was left overnight for ethanol evaporation, in the dark, and under reduced pressure. After 24 h, a viscous liquid resulted, consisting of the cannabinoid and the polymer (CBDm for CBD-PF127 mixture and CBGm for CBG-PF127 mixtures). The viscous liquid was added to distilled water (0.5 mL) and was sonicated for 2 min. After sonication, an opaque suspension of nanoparticles was formed. In method B, the solvent displacement approach was followed [32]; accurate amounts of CBD or CBG (5 mg) and PF127 (5 mg) were weighed and solubilized in 99% ethanol (50 μL) while being vortexed. Subsequently, 10 μL of the ethanolic solution were injected into the aqueous phase (1 mL distilled water) and subjected to sonication in an ice bath (SONICS, Vibracell^TM^, Newtown, CT, USA) for a total period of 30 s (10 s on, 5 s off circle). Upon sonication, an opaque suspension of nanoparticles was formed. Ethanol was selected as a solvent that is water-miscible, volatile, and common for cannabinoids and the polymer [34]. During the experiments, samples were kept at room temperature and protected from light.

### 2.3. Development of the 3D-Printed Films

Three film formulations with different concentrations in cannabinoid suspensions were prepared (M1, M2, and M3, Table 1). SA and calcium chloride were dissolved separately in double-distilled water under magnetic stirring at room temperature for 24 h until homogeneous viscous solutions were formed. The samples were stored at 4 °C, as lower temperatures reduce Ca^2+^ reactivity. Additionally, 3 mL of 10% SA solution were mixed with 2 mL of cannabinoid suspensions (4, 8, or 12 mg/mL) and 0.4 mL of 10% calcium chloride solution to obtain the 3D-printable inks. The ingredients concentrations in the final mixture are mentioned in Table 1. The addition of calcium chloride promotes the formation of a viscous gel due to cross-linking. The prepared 3D-printable inks were loaded to an extrusion-based 3D printer (CELLINK^®^ Inkredible, Gothenburg, Sweden), and three-layer rectangular films (2 cm diameter) were printed. After 3D-printing, free-standing films were produced by immersing them in 10% calcium chloride solution for 2 min.

### 2.4. Differential Scanning Calorimetry (DSC)

The melting temperature, glass transition temperature, and the crystalline behavior of CBDm, CBGm, and raw materials (PF127, CBD, and CBG) were determined using DSC. Pre-weighed samples (5 mg) were placed into a perforated aluminum pan and were heated from 20–120 °C. The thermograms were recorded at a heating rate of 10 °C/min using a DSC 204 F1 Phoenix heat-flux Differential Scanning Calorimeter (NETZSCH, Selb, Germany) under an inert atmosphere (nitrogen purge gas, flow rate 70 mL/min). The instrument was calibrated using indium standards.

### 2.5. Thermo-Gravimetric Analysis (TGA)

The TGA analysis of CBDm, CBGm, PF127, CBD, and CBG was conducted using a Shimadzu TGA-50 instrument (Tokyo, Japan). Pre-weighed samples (5 mg) were heated from 30 to 300 °C at a heating rate of 10 °C/min under an inert atmosphere.

### 2.6. Fourier-Transform Infra-Red (FTIR)

Using an ATR-FTIR spectrometer (IR Prestige-21, Shimadzu, Japan), FTIR analysis was performed on nanoparticle suspensions of different drug/polymer ratios, on 3D-printed films right after printing and 48 h drying in ambient conditions, and on raw materials (SA, PF127, CBD, CBG, and calcium chloride) accordingly. A broad scan of the samples was performed from 500 cm^−1^ to 4000 cm^−1^ with a resolution of 4 cm^−1^. A total of 64 spectra was averaged to reduce noise. The spectra were recorded in absorbance mode at room temperature. The process of the spectral data was completed with the commercially available software IR Solutions (Shimadzu, Tokyo, Japan)

### 2.7. Particle Size and Polydispersity Index (PDI)

Initially, method A was used to determine the optimum cannabinoid/PF127 ratio for forming nanoparticles with the preferred characteristics (small size and PDI). Suspensions of 1:1 ratios of cannabinoid: PF127 were prepared with method A. The selection of the preferred ratio was followed by comparison tests on suspensions with different concentrations in nanoparticles (0.4%, 0.8%, and 4% *w*/*v* in water) prepared with method A. A comparison test was conducted between the two preparation methods (A and B). The average particle size and PDI values were determined by Dynamic Light Scattering (DLS) with a Zetasizer (Malvern Panalytical, Malvern, UK). The measurements were performed at 25 °C.

### 2.8. Preliminary Characterization of the Films

The pH of the films was measured by pressing pehametric test strips on their surface. In addition, their weight and average thickness were determined using a 0–25 mm (±0.01 mm) Vernier Caliper at five randomly scattered points for each film. The measurements were carried out in triplicate. The results were expressed by the mean of the measurements ± standard deviation (SD).

### 2.9. Water Retention Capacity and Porosity

For the calculation of water retention capacity and porosity of the films, samples were printed, cross-linked with calcium chloride, and their weight was measured. Each sample was immersed in a falcon tube that contained PBS at room temperature. Every 10 min for 1 h, and then, once after 24 h, specimens were withdrawn from the PBS. After removing excess water by gently blotting the films’ surface, their weight was measured. Τhe test was carried out in triplicate. Their weight variation as a function of time is given from Equation (1):

(1) $$W\;\left(\%\right)=\frac{W_{t}}{W_{0}}\times 100,$$

where W% is the % film weight variation, W_t_ is the film weight at a certain time, and W_0_ is the initial film weight.

Total porosity is calculated using Equation (2), where ρ_film_ is the density of the 3D-printed film and ρ_mixture_ is the density of the gel before 3D printing [42]. The density of a mixture (P_mix_) is given using Equation (3), where ρ_ν_ is the density of each substance and Χ_ν_ is the molecular ratio of every substance.

(2) $$\Pi \;\left(\%\right)=1-\frac{\rho_{\mathrm{film}}}{\rho_{\mathrm{mixture}}\;}\times 100$$

Ρ_mix._ = X_ν_ × ρ_ν_ + X_ν+1_ × ρ_ν__+1_ + … (3)

### 2.10. Water Loss Rate

The evaporation of water through the film was determined by periodic weighing. Rectangular films were printed, cross-linked and placed in Petri dishes for 72 h in room environment conditions. Every 15 min for 4 h and once after 24 h and 72 h, specimens were weighed, and the weight loss as a function of time plot was constructed. Weight loss was indicative of the loss of water molecules. The film’s relative water weight (the water weight of each film divided by its initial water weight) with the time of the measurement can be calculated from Equation (4):

(4) $$W\;(\%)=\frac{W_{\mathrm{wt}}}{W_{\mathrm{wo}}}\times 100,$$

where W_wt_ is the water weight in the film every t and W_w0_ the initial weight of water in the film.

### 2.11. Optical Microscopy

Assessment of the membrane surface’s texture and morphology is very important for ensuring the uniform release of the drug and distribution on the skin. To analyse these parameters, samples were printed and observed under an optical microscope (Celestron MicroDirect 1080 p HD Handheld Digital Microscope, Celestron, Torrance, CA, USA). Photographs of the films were captured at a magnification of 220×.

### 2.12. Mechanical Tests

Shear resistance of the loaded and non-loaded 3D printed films (50 × 50 mm) was evaluated using a Testometric testing machine (Rochdale, UK) according to ASTM D 732-17. A punch tool (15 mm flat design cylindrical puncture head) was used to create a hole in each sample. A die cavity with an average diameter of 15 mm was achieved in each sample by shearing it against two pieces of metal. Shear tests were conducted in triplicate with a crosshead speed of 5 mm/min at ambient temperature. The shear performance of the specimens was determined in terms of shear strength, energy to shear, and ductility. The shear test is an appropriate method to investigate the mechanical properties of resilient materials that may stretch excessively and have ambiguous results when tested in the tensile mode. The maximum force, the area under the force-displacement curve, and the ultimate displacement of the punch probe are the most important factors. From these data, shear strength and energy to shear were calculated. The Young’s modulus or elongation to shear is impossible to be calculated with this methodology [43]. Shear strength is calculated based on Equation (5):

Shear strength (N/mm^2^) = F/Ac (5)

where F is the load required to shear the film and Ac is the area of the sheared edge, which shall be taken as the product of the specimen thickness by the circumference of the punch. The equation for energy to shear is similar to that of a tensile test, except for the calculation of the volume term. Hence, the energy to shear per unit volume is calculated by:

Energy to shear (J/cm^3^) = As/Vc (6)

where As is the area under the load—displacement curve of the shear tests and Vc is the volume of the die cavity of the film holder. The displacement of the shear punch until fracture has been normalized by the initial thickness of each specimen and considered as ductility.

### 2.13. 3D-Printing Shape Fidelity Assessment

**Figure 2:**
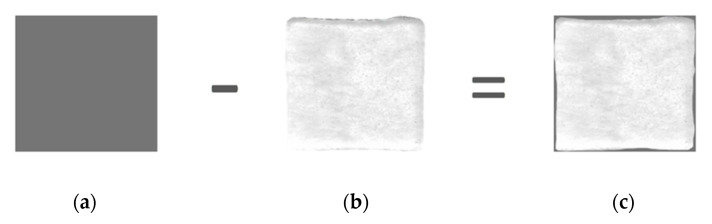
(**a**) Theoretical film dimensions, (**b**) 3D-printed film dimensions immediately after printing and (**c**) their difference as calculated by Equation (7).

The shape fidelity assessment was based on the quantification of the difference between fabricated 3D-printed film and their expected dimensions [6]. The 3D-printed films (2 × 2 cm) were prepared and scanned with a conventional 2D scanner (HP DesckJet 2630, Hewlett-Packard, Palo Alto, CA, USA). Their dimensions were compared to a control frame (2 × 2 cm) that was designed in Adobe Photoshop (Adobe Inc., San Jose, CA, USA) (Figure 2). The deviation of the expected shape is expressed as in Equation (7):

(7) $$Ε\;\left(\%\right)=\frac{\left(\mathrm{Ac}-\mathrm{Ap}\right)}{\mathrm{Ap}}\times 100,$$

where Ac and Ap are the projection areas (in pixels) of the control frame and the 3D-printed film respectively.

### 2.14. Quantitative Analysis of Cannabinoids

The quantitative analysis of CBD and CBG was performed by High-Performance Liquid Chromatography (HPLC). The analytical conditions were adopted and modified from the literature [44]. The HPLC system consisted of a pump (LC-10AD VP) that was maintained at room temperature, an auto-sampler (SIL-20A HT), and an Ultraviolet-Visible detector (SPD-10AVP) (Shimadzu, Kyoto, Japan). The analysis was performed using a reversed-phase Discovery HS C18 (15 cm × 4.6 mm, 3 μm) column (Sigma-Aldrich, Merck, KGaA, Darmstadt, Germany). The mobile phase was a mixture of acetonitrile (A) phosphate buffer (B) (KH_2_PO_4_, 0.0126 M, pH 5.0, A:B 80:20 *v*/*v*). The mobile phase was degassed under vacuum (20 min), sonicated (10 min), and filtered (0.45 μm pore size, Whatman^®^, Sigma-Aldrich) prior to the analysis. The injection volume was 30 μL, and the flow rate was maintained at 1 mL min^−1^. The retention time of CBD was 5.5 min and of CBG was 5.0 min. The quantification of the active compounds was performed by ultraviolet-visible detection at 230 nm. The linearity of the method was investigated for both compounds by data collection at different concentrations in the range of 1–100 μg/mL (R^2^ ≥ 0.9999). All samples and standards were analyzed in triplicate. Prior to the quantification with HPLC, the prepared nanoparticle suspensions were centrifuged (4500 rcf, 10 min), the insoluble fraction of cannabinoids precipitated, and the supernatant was filtered through a 0.45μm poly-vinylidene fluoride (PVDF) filter.

### 2.15. In Vitro Release Study of Cannabinoids from Loaded Films

To evaluate the CBD and CBG release profiles from loaded films, samples of three different concentrations (4 mg/mL, 8 mg/mL, and 12 mg/mL) were prepared with method B. The samples were placed in glass vessels filled with 40 mL of phosphate buffer saline (PBS; sodium chloride, 8 g/L; potassium chloride, 0.2 g/L; sodium phosphate di-basic, 1.44 g/L; potassium phosphate monobasic, 0.24 g/L; pH 7.4;) and 0.5% SLS. The release study was performed at 37 °C with continuous stirring for 24 h and sampling at regular time intervals (0.5, 1, 2, 3, 4, 5, 6, 7, 8, 24 h) [45,46]. At predetermined time points, aliquots (1 mL) were removed from vessels and were immediately replaced with an equal amount of preheated PBS [37,47,48]. All collected samples were mixed with 1 mL methanol, filtered through 0.45 μm polyvinylidene fluoride (PVDF) filters, and analyzed with HPLC.

### 2.16. Entrapment Efficiency and Drug Loading

Drug entrapment efficiency (EE) and drug loading capacity (DL) refer to the amount (%) of drug entrapped in PF127. Pre-weighed nanoparticle suspension samples were solubilized in a mixture of acetonitrile:water 80:20 (1 mL). After filtration and suitable dilutions, the samples were analyzed by HPLC, and the EE and DL were calculated according to Equations (8) and (9):

(8) $$\mathrm{DL}\;\left(\%\right)=\frac{W_{\mathrm{drug}}}{W_{0}}\times 100$$

(9) $$\mathrm{EE}\;\left(\%\right)=\frac{W_{\mathrm{drug}}}{W_{\mathrm{loaded}}}\times 100,$$

where W_drug_ is the weight of the drug encapsulated in the solid suspension, W_0_ is the initial weight of the drug used in the formulation, and W_loaded_ is the weight of the nanoparticle suspensions samples. The results are presented as the average value of three independent measurements for each sample.

### 2.17. Cell Culture

HaCaT cell line (passage 9–15) was implemented in the present study to investigate the in vitro wound healing and cell viability after treatment with the produced CBD and CBG nanoparticles. HaCaT cells were cultured in DMEM supplemented with 10% (*w*/*v*) FBS, 1% (*v*/*v*) penicillin-streptomycin, and 1% (*v*/*v*) Glutamax. The cells were incubated at 37 °C in a 5% *v*/*v* CO_2_ humidified atmosphere.

### 2.18. Cell Viability—MTT Assay

Cell viability was studied using the MTT assay. HaCaT cells were seeded in 96-well plates at a density of 10^4^ cells/well. After 24 h incubation, the cells were treated with three different concentrations of CBD and CBG nanoparticles, 0.1, 1, and 5 mg/mL. After 24 h and 48 h, the culture medium was then removed, and the cells were rinsed with PBS. MTT solution (5 mg/mL) was then added to the cells; they were further incubated at 37 °C. After 3 h the MTT was removed, and DMSO was added to dissolve the formazan crystals. The well plate was subjected to mild agitation for 15 min, and the absorption of each well was measured at 540 nm. Equation (10) was used to calculate the cell viability.

Cell Viability (%) = 100 × (A_S_ − A_B_)/(A_C_ − A_B_) (10)

where A_S_, A_B_ A_C_, and are the absorbance values of the sample well, the blank well and the control well, respectively.

### 2.19. Cell Scratch Assay (Wound Healing)

For the assessment of the wound-healing activity of the CBD and CBG nanoparticles, a cell scratch assay was selected as a convenient, well-developed, and inexpensive in vitro method for the analysis of cell migration. HaCaT cells were seeded into 6-well plates (2 × 10^5^ cells/well), incubated at 37 °C, and allowed to grow for 48 h in the culture medium. In each well, the cell monolayer was scraped by two straight perpendicular scratches that were created with a sterile pipette tip. The scratch was used as a reference point. After rinsing with PBS, the cells were incubated with three concentrations of nanoparticles (0.1, 1, and 5 mg/mL) and a serum-free medium. The cell migration and quantity of cells in the scratch area in each well were monitored under a microscope, and the images were recorded at 0, 6, and 12 h. Wound closure was calculated according to Equation (11):

Relative Wound Closure (%) = 100 × (A_i_ − A_f_)/A_i_, (11)

where A_i_ and A_f_ are the initial and final cell-free area of the simulated wound, respectively.

The wound width before and after incubation with the nanoparticles was quantified manually. Then, 50 width measurements were taken between the edges of the scratch and their average was calculated. The wound area was calculated by tracing the areas lacking cells in the captured images using ImageJ software (NIH, Bethesda, MD, USA).

### 2.20. Antibacterial Activity In Vitro

The antibacterial activity of the cannabigerol (CBG) and cannabidiol (CBD) formulations were evaluated via the agar diffusion method [49,50] using filter paper discs and well-characterized clinical isolates of bacteria. In brief, colonies of *E. coli* or *S. aureus* isolated from nutrient agar were inoculated to the Nutrient broth and incubated at 37 °C for 18–24 h, while a *Bacillus* spp. was isolated from Mueller Hinton agar plates and grown in Mueller Hinton (MH) broth in the same fashion. The bacterial concentration of each overnight culture was adjusted to McFarland 0.5 standard, and 100 μL inoculum was spread evenly on MH agar plates, following which filter paper discs of 6 mm diameter loaded with 5 μL of each sample were placed. The plates were incubated at 37 °C for 18–24 h, and the diameter of each growth inhibition zone was measured and compared with that of a control sample. Each time, a neomycin disc (---) was used as a positive control. All disc diffusion tests were repeated three times, and the mean values were calculated.

### 2.21. Statistical Analysis

All experiments were conducted in triplicates, and statistical analysis for different samples was performed using OriginLab v9.0.0 software (Originlab Corporation, Wellesley Hills, MA, USA). All results are expressed as mean ± standard deviation (±STD). One-way ANOVA was used for the determination of the significant difference at which statistical significance was reported when the *p*-value was less than 0.05. For the statistical analysis, SPSS software version 16.0 (SPSS Inc., Chicago, IL, USA) was employed.

## 3. Results

### 3.1. Development of Cannabinoid Nanoparticles

Nanoparticles below 500 nm exhibit unique properties due to their high surface area-to-volume ratio. They ensure direct contact with the stratum corneum and the skin appendages, and they improve dermal permeation. The ratio of the polymer and the active ingredient has been proven to affect the formation and characteristics of the nanoparticles [35]. Thus, different studies were conducted to determine the formulation procedure that results in the optimum nanoparticle size and PDI values. Eleven different ratios of PF127 and cannabinoid were developed with method A, and their size was evaluated by means of DLS. Results showed that samples with a 1:1 ratio presented the best PDI values (0.29 ± 0.06 and 0.22 ± 0.07) and particle sizes (197 ± 28.6 nm and 229 ± 58 nm) for CBD and CBG, respectively. Both CBD and CBG formulations showed uniform nano-sized dimensions with diameters smaller than 200 nm and small PDIs. For the selected ratio (1:1), additional measurements were conducted on the dilution of CBD and CBG in different water volumes (0.4, 0.8, and 4% *w*/*v*). For both cannabinoids, the best results were obtained at 4% *w*/*v* concentration. Finally, Method A and Method B were compared for the 4% *w*/*v* concentration and 1:1 ratio. Both preparation methods resulted in nanoparticles with comparable sizes and PDI values. The results of the DLS analysis are presented in Table 2. Method A nanoparticles presented a better PDI value (0.203 and 0.202 for CBD and CBG, respectively) and were used to prepare the 3D printed films.

### 3.2. Evaluation of 3D Printed Films

Free-standing SA films with cannabinoids at three different concentrations (6, 8, and 12 mg/mL) were successfully prepared using calcium chloride as a crosslinker. In all formulations, the optimum cannabinoid:PF127 (1:1) ratio was employed. The cross-linking structure is explained by the egg-box model and is based on the formation of links between the carboxylate groups of SA and the calcium ions [51,52]. After 3D printing, the visual examination of the films did not show significant differences between the printed samples. The produced films had a fair level of transparency, enabling the possible wound monitoring during the healing process without film removal. All films were smooth and evenly shaped with an average thickness of 0.8 ± 0.002 mm and weight of 0.73 ± 0.150 g. However, minor 3D-printing-related defects could be noticed, as shown in Figure 4. The measurement of film thickness and weight is necessary as they are directly related to the amount of drug in the film. In addition, the deviation of the printed film when compared to the computed designed one was found to be 7.51% ± 0.150. The films’ porosity was 11.00 ± 0.002 (ρ_mixture_ = 1.05 g/cm^3^, ρ_patch_ = 1.04 g/cm^3^). Thus, the limited variation of these parameters is indicative of the consistent amount of drug in the formulated films without significant deviation (Table 3).

#### 3.2.1. pH Determination

Studies have shown that pH has a major role in the wound healing process, as it affects the biochemical reactions that take place. Skin pH ranges between 4.8–6.0, while for chronic wounds between 7.2–8.9 [53]. For chronic ulcers, it ranges between 5.7 for category I and 6.9 for category II to 7.6 for category III, presenting a wound severity-dependent pH value. Studies have presented that the pH of wounds, usually higher than 7.4, delays the healing procedure [53]. In addition, according to research, acidity has a significant influence on fibroblast proliferation and a positive influence on healing [54]. Thus, wound dressings should be on the slightly acidic side. The pH value of the fabricated loaded films was 6–7 for all formulations proving their adequacy for the injured skin.

#### 3.2.2. Film Water Loss Rate

**Figure 3:**
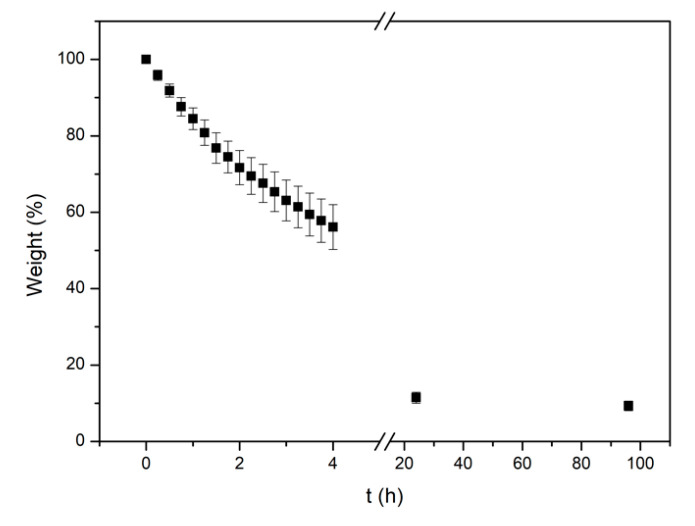
The films relative water weight with the time of the measurement.

The water loss rate was calculated from Equation (4) and is presented in Figure 3. In the first 4, 24, and 96 h, 44%, 88%, and 90% of water, respectively, had evaporated. It is prevalent that a significant water loss takes place in the first 24 h resulting in film dehydration. This could be prevented if an additional top layer was added to provide a moist internal environment for longer and further contribute to the healing process.

#### 3.2.3. Film Surface Observation

**Figure 4:**
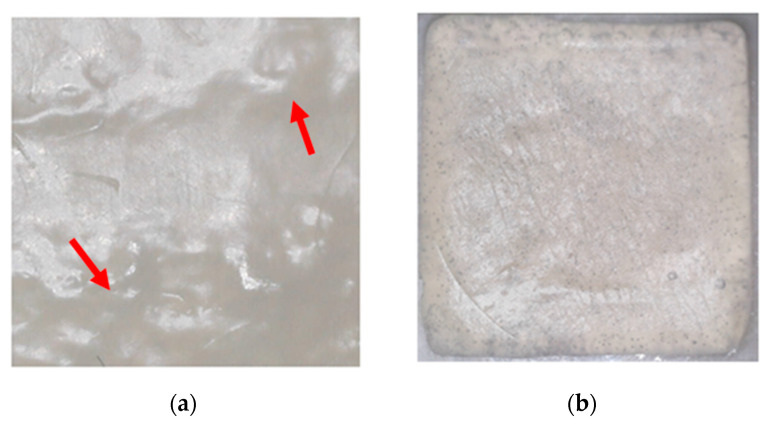
(**a**) Optical microscopy image of the non-loaded 3D-printed film and (**b**) non-loaded 3D-printed film after cross-linking with 10% CaCl_2_ solution. Red arrows show the defects on the surface of the film.

For optimal distribution of the drug on the skin, the film’s surface should be homogenous. The morphology of the 3D-printed films without nanoparticles was observed under an optical microscope. The observed image is presented in Figure 4, which reveals the presence of printing-related defects on the surface of the film. These defects are characterized by the presence of voids formed during the printing process, which gradually decrease in size and result in bubble-like formations. The presence of the larger coagulants could be further explained by irregular topical cross-linking. Uniform surface morphology is linked with the uniform distribution of the drug throughout the film and, thus, the uniform release of the drug on the skin. However, cross-links in the current films are formed in an instantaneous way that prevents homogenous structures from being formed. The film is expected to consist of regions of high and low cross-link density that are not even throughout the film [55]. The particles are expected to be uniformly distributed into the ink formulation (prior to cross-linking) and consequently into the film formulation. The uniform distribution of the particles into the films ensures the release profiles of the APIs. Nevertheless, it is also expected, based on the existing literature, that non-uniform regions are going to be present throughout the bulk of the film due to different cross-linked densities [41,56,57]. These differences were expected, and they are consistent with every printed film. The resulting SA gels are highly dependent on the gelation rate and the way of adding calcium ions to the mixture [51]. Internal gelation results in gel structures with higher homogeneity but lower strength and looser networks. The external gelation method, which was employed in the present work, creates gels with lower homogeneity but increased strength. Additionally, it is a fast technique that has been widely used in the last years for the micro-encapsulation of active ingredients [58]. Thus, some defects could be attributed to the non-automated mixing that did not provide calcium ions the same chances of contact with the binding sites of SA to create uniform networks.

#### 3.2.4. Water Retention Capacity and Porosity

Figure S1 (Supplementary Material) demonstrates the variation of the films’ relative weight (the weight of each film divided by its initial weight) within the timeframe of 24 h. After immersing the films in PBS for a certain period, the membranes’ microstructure begins to change. As PBS enters the polymer matrix, the chains begin to relax and turn into oligomers and monomers [2,59]. This promotes the diffusion of a larger amount of PBS, and the samples begin to gradually dissolve. The measurement at 24 h exhibited a high standard deviation which is possibly the result of the unordered decomposition of the samples. This was visually confirmed during the experiment. Printed films did not present fluid uptake efficiency as their weight was steadily decreasing. However, they might be able to enhance wound healing by providing the wound with the desired humidity. In addition, they could have hemostatic properties owing to the ion exchange between the calcium ions of the alginate network and sodium ions of the wound exudates [60]. The porosity, as calculated from Equation (2), was 11 ± 0.0024.

#### 3.2.5. Mechanical Tests

Shear tests by the punch-type tool were implemented to determine the mechanical properties of the films.

**Figure 5:**
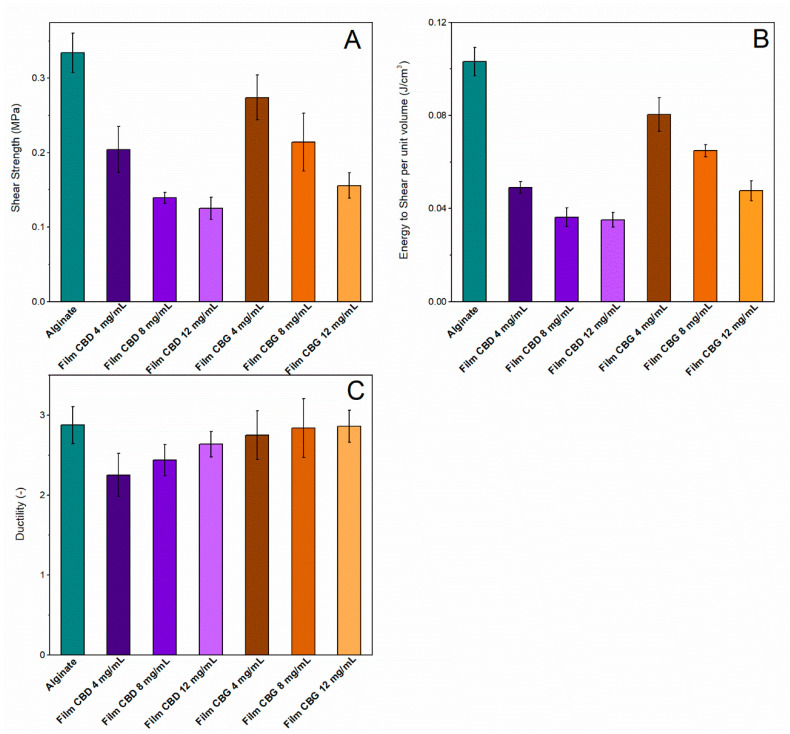
(**A**) Shear strength, (**B**) Energy shear per unit volume and (**C**) Ductility of the loaded and unloaded films.

The shear test data revealed that non-loaded alginate specimens have the highest shear strength, as shown in Figure 5. The addition of CBD revealed a deterioration of the shear resistance. The same phenomenon occurred with CBG in alginate specimens. This deterioration is shown to be similar for both CBD and CBG loadings (*p* < 0.05) (Figure 5A). The alginate/CBD 4 mg/mL specimens demonstrated the highest shear strength compared to the other CBD-loaded films (*p* < 0.05). The alginate/CBG 4 mg/mL specimens demonstrated the highest shear strength compared to all loaded films (*p* < 0.05). Additionally, the results show that there is a moderate increase in the ductility for alginate/CBG samples, with an increasing concentration of CBG, as shown in Figure 5C. Also, the alginate/CBD 4 mg/mL specimens had the lowest elongation at break (*p* < 0.05), as shown in Figure 5C. For the CBG samples, it seems that the ductility has been only mildly affected.

The values of energy to shear fracture are shown in Figure 5B. The neat alginate specimens were the most resistant to shear and required the greatest amount of energy to shear fracture. A similar phenomenon occurred for the calculation of shear strength, following the same trend. Alginate/CBG 4 mg/mL samples were considered as the specimens with the highest energy to shear among the loaded specimens. This can be attributed to a combination of their high shear strength and relatively high elongation to fracture. Therefore, alginate/CBD 12 mg/mL specimens were the least resistant to shear fracture by punch tool (*p* < 0.05).

### 3.3. Fourier-Transform Infra-Red (FTIR)

**Figure 6:**
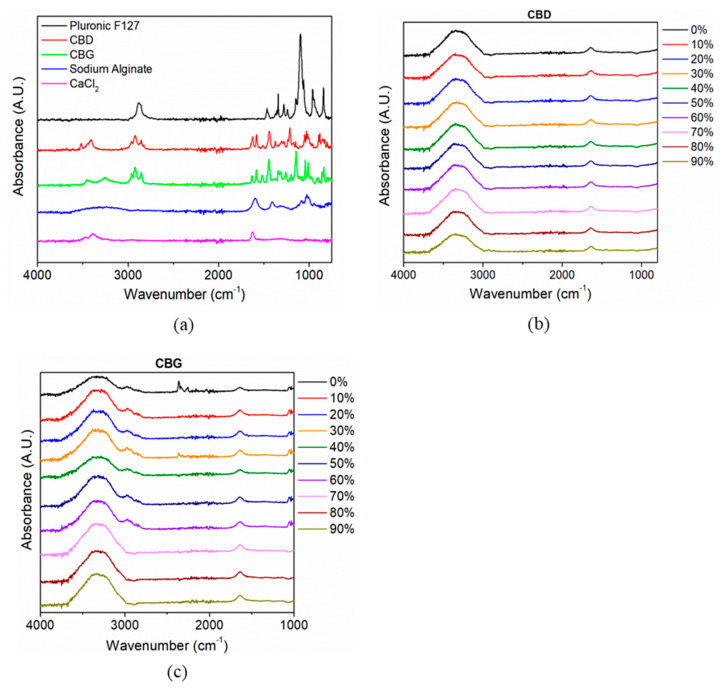
FT-IR spectra of (**a**) raw materials, (**b**) CBD dispersion and (**c**) CBG dispersion.

FT-IR method was employed to investigate the appearance or modification of the position of characteristic peaks that could be attributed to possible interactions between the different groups of interest. Thus, it was used for the evaluation of the chemical composition of the films and the possible interactions between SA and calcium chloride (Figure 6). The chains of SA tend to form 3D structures with calcium chloride because of the carboxylate groups [52]. Sodium alginate spectrum (Figure 6a) exhibits a characteristic broad absorption band at 3000–3600 cm^−1^, which can be due to the vibration of the –OH group, at 1600 cm^−1^ due to the asymmetric stretching vibration of COO^−^ groups, at 1400 cm^−1^ due to the symmetric stretching vibration of COO^−^ groups, and at 1050 cm^−1^ due to the elongation of CO- groups [61].

FTIR analysis can also be used for studying the molecular interactions and chemical structure of CBD and CBG suspensions. The FTIR spectra for different ratios of CBD/PF127 and of CBG/PF127 are shown in Figure 6b,c, respectively. For both cannabinoids, two distinct peaks can be observed at 1580 cm^−1^ and 1620 cm^−1^, possibly due to the C=C stretching vibration of the compounds [7]. If nanoparticles are being formed, the high-intensity peaks of pure CBD and CBG are expected to shift or disappear as their successful formation would result in the restriction of the above-mentioned groups. The absence of the characteristic CBD and CBG bands in the spectra of their suspensions indicates an association of the hydrophobic double bond moiety of CBD with the hydrophobic interior of PF127 [7]. In addition, it could also suggest the absence of free drug on the surface of the polymer as cannabinoids may have entirely or partly been entrapped in the hydrophobic center of the micelle-like structure. The drug incorporation into that structure is also expressed by the high similarity of the CBD and CBG spectra when they are in nanoparticle form. Possible intermolecular interactions could also be present but too small to be detected with FTIR.

### 3.4. Differential Scanning Calorimetry (DSC)

**Figure 7:**
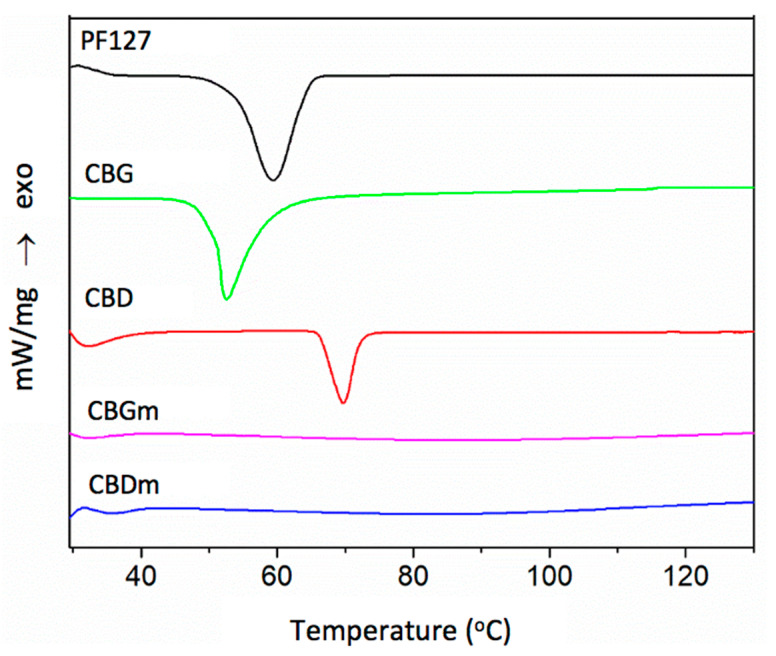
DSC thermograms of PF127, CBG, CBD, CBGm and GBDm.

DSC analysis is a common method for studying the physical state of particles. Thus, it was used to determine the quantity of heat absorbed or released when CBDm and CBGm undergo physical or chemical changes. The thermograms obtained by the analysis of CBDm and CBGm as well as of raw materials (CBD, CBG, and PF127) are displayed in Figure 7. Regarding the raw materials, the characteristic sharp peaks located at 71 °C, 47 °C, and 59 °C indicate the melting points of CBD, CBG, and PF127, respectively [44,59,62]. For CBDm and CBGm the endothermic peaks are wider and can be observed at lower temperatures (35 °C and 32 °C, respectively). The DSC curves for CBDm and CBGm do not present the sharp endothermal events that occur in the thermograms of CBD and CBG, reflecting the potential transition of the cannabinoids from crystalline solids into an amorphous state or the molecular dispersion of the cannabinoids in the PF127 matrix [62].

### 3.5. Thermogravimetric Analysis (TGA)

**Figure 8:**
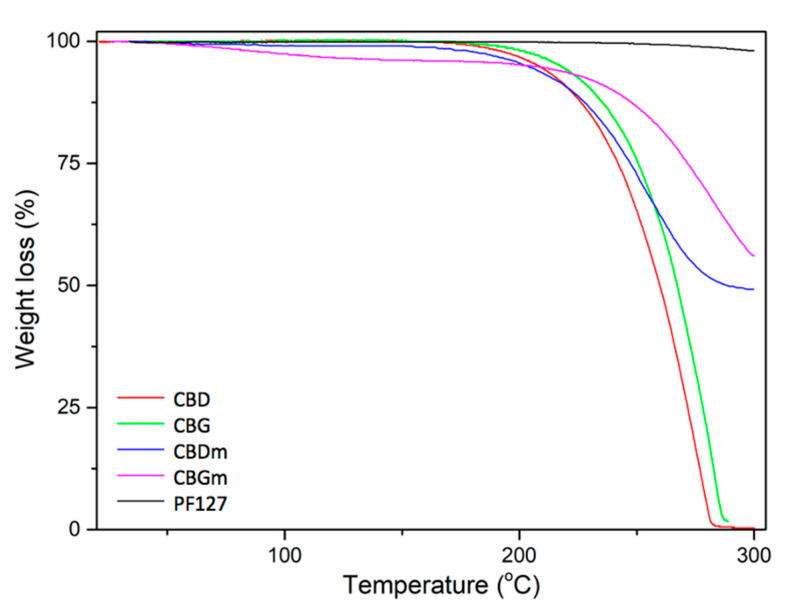
TGA thermograms of CBD, CBG, CBDm, CBGm, and PF127.

TGA analysis was performed to determine the thermal properties of the samples because they provide useful information for the characterization of materials. In Figure 8, thermograms of CBDm and CBGm, CBD, CBG, and PF127 in the range of 30–300 °C are presented. For CBG, decomposition starts around 200 °C and is completed at 230 °C. For CBGm, the degradation starts around 230 °C, while 45% weight loss is observed at 300 °C. The thermal decomposition of CBD starts around 230 °C and is completed at 280 °C. For CBDm, degradation starts around 190 °C, and up to 300 °C its weight is reduced by 50%. These results further confirm the successful formation of nanoparticles and the increased thermal stability of cannabinoids in the hydrophobic center of the new structures. The high thermal stability of PF127 possibly increased the thermal stability of cannabinoids in the nanoparticles. In the temperature range of these thermograms, no other significant thermal events are present. Therefore, these results, combined with the findings of FTIR and DSC, indicate that CBD and CBG nanoparticles have successfully formed.

### 3.6. In Vitro Release of CBD and CBG from the Films

**Figure 9:**
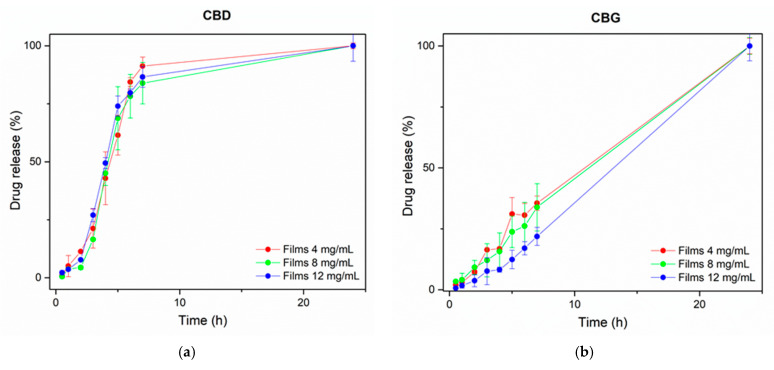
The release profile of (**a**) CBD and (**b**) CBG from the 3D-printed film in PBS pH 7.4 at 37 °C. Each point represents the mean ± SD, n = 3.

The in vitro release profile of the cannabinoids from the films in three different concentrations is depicted in Figure 9. CBD and CBG were released from the films within 10 h and 24 h, respectively. Prolonged inflammation is the main cause of a wound. Thus a prolonged release of the drug is desirable for the proper wound healing process [63]. Increasing concentrations of particles resulted in a more prolonged release as seen in Figure 9b for CBG. Films 4, 8, and 12 mg/mL released 35%, 33%, and 21% of the drug within 7 h, respectively. Thus, a prolonged release profile is observed for the higher concentration of particles. The release data collected from the CBD profile were optimally fitted to Korsmeyer—Peppas model (R^2^ ≥ 0.9269, k = 0.007, n = 1.568), while the release data from the CBG results were optimally fitted to the Zero order model (R^2^ ≥ 0.9672, k = 3.886).

### 3.7. Drug Loading and Entrapment Efficiency of Nanoparticles

To determine the amount of CBD or CBG that was entrapped inside the nanoparticles, the DL and EE were calculated. The DL and EE values obtained were 98.25 ± 1.20% and 99.35 ± 2.35% for CBG and 97.58 ± 2.39% and 98.39 ± 1.87% for CBG, respectively. Τhese results are considered very satisfactory as they prove high drug loading and encapsulation efficiency.

### 3.8. Cell Viability—MTT Assay

**Figure 10:**
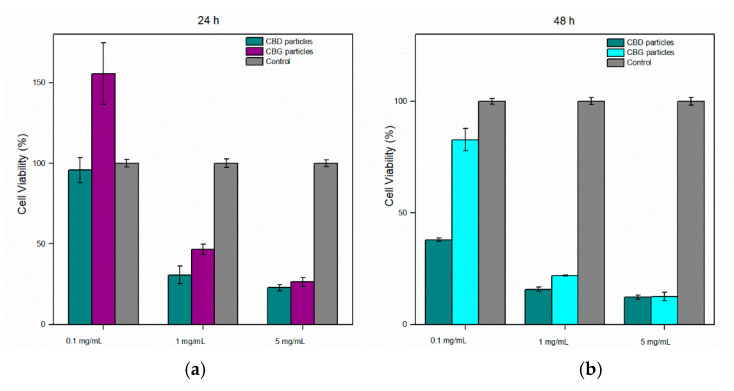
Cell viability of HaCaT cells after exposure to various concentrations of CBD and CBG particles (**a**) after 24 h and (**b**) after 48 h, as measured by the MTT assay.

Cell viability upon incubation with three different concentrations of CBD and CBG particles is illustrated in Figure 10. Higher concentrations (1 or 5 mg/mL) of both cannabinoids resulted in poor cell viability (<50%). In addition, 5 mg/mL was a high concentration that was expected to result in a cytotoxic effect; the latter might be attributed to high concentrations of PluronicF127 tested [64]. Nanoparticles in 0.1 mg/mL did not exhibit cytotoxicity in the first 24 h, whereas CBG particles (0.1 mg/mL) had no cytotoxic effect even after 48 h. Consequently, from these results, CBD and CBG nanoparticles’ cytotoxicity is found to be related to their concentration and the time of exposure.

### 3.9. Cell Scratch Assay (In Vitro Wound Healing)

**Figure 11:**
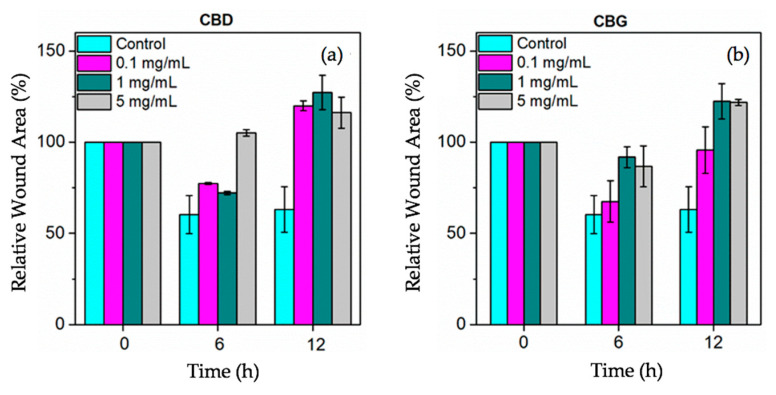
Relative Wound Area calculated by the in vitro wound-healing assay for (**a**) CBD and (**b**) CBG nanoparticles in various concentrations (0.1, 1 or 5 mg/mL).

**Figure 12:**
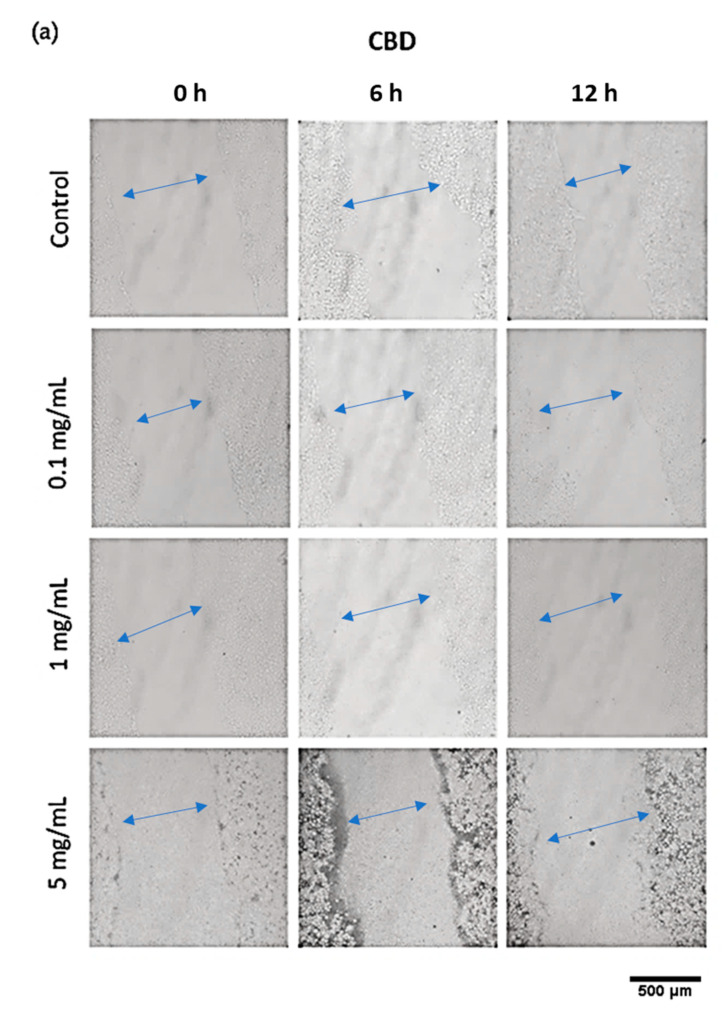

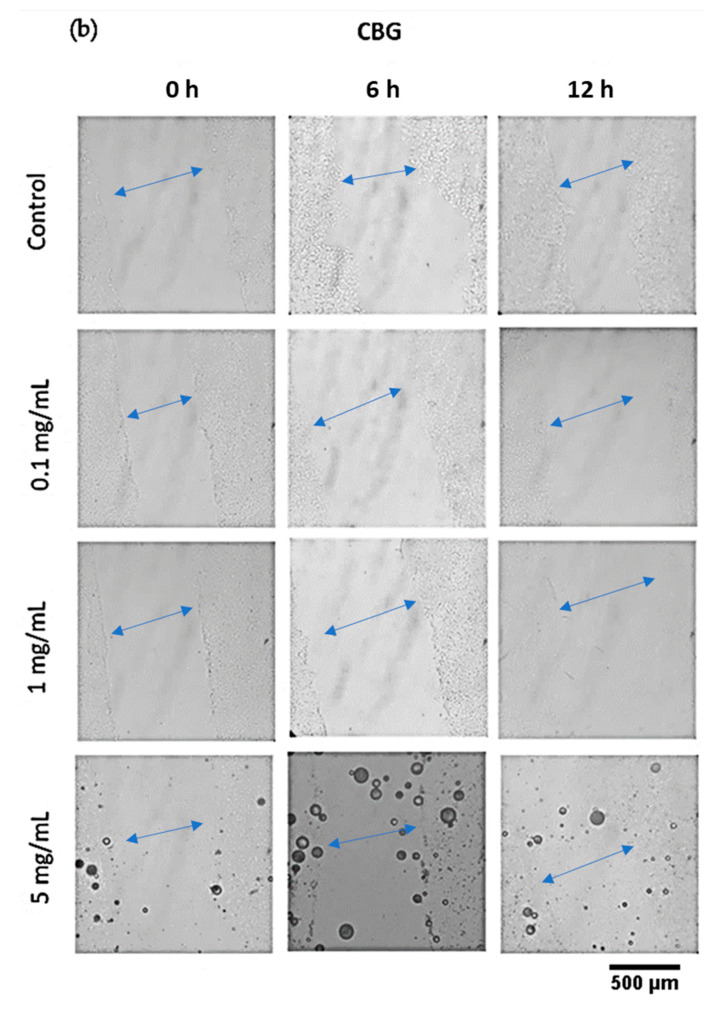
Time-Lapse of the in vitro wound healing process for various concentrations (0.1, 1 and 5 mg/mL) of (**a**) CBD and (**b**) CBG nanoparticles in the cell culture medium (100× magnification). Blue arrows show the scratch surface in the every time point.

The assessment of the wound-healing potential of CBD and CBG nanoparticles was conducted with a cell scratch assay for various nanoparticle concentrations (0.1, 1, and 5 mg/mL). The wound was simulated by generating two perpendiculars in each well. The wound closure was monitored over a period of 12 h in 6-h time intervals. Figure 11 presents the relative wound area (%) as calculated by the in vitro wound healing assay. Figure 12 shows a time-lapse of the actual wound healing process. As depicted in Figure 11, in the first 6 h, the relative wound area (%) decreased for both cannabinoids and for all concentrations. However, in the following, for all concentrations, the relative wound areas are higher than the initial ones. CBD has been tested as an oral wound healing constituent. This study revealed that CBD has an anti-inflammatory effect on the wound, but it is not sufficient to promote the wound healing process [65]. Another study indicates that short-term exposure to CBD may promote the wound healing process on gingival fibroblasts [66]. These findings are in accordance with our study showing that short-term exposure to CBD nanoparticles decreased the wound area on HaCaT cells. CBG nanoparticles in concentration 0.1 mg/mL were an exception and presented slightly decreased wound area, even after 12 h. These results could be connected to the ones presented in the MTT assay, according to which cytotoxicity increased with the time of exposure and concentration. This exception may also be attributed to the fact that CBG has better anti-inflammatory and antioxidant properties than CBD and has a better effect on skin health-promoting activities [67]. These results showed that the CBD and CBG nanoparticles do not show strongly in vitro wound healing properties but only a short-term effect on the healing process. Complementary cell studies enrolling other types of cell lines (e.g., dermal fibroblasts) are needed to elucidate the mechanism of action of these films nanomaterials [68]. Further exploration is needed to confirm the wound healing effect of these substances.

### 3.10. Evaluation of In Vitro Antibacterial Properties

Growth inhibition by the prepared CBG or CBD-loaded formulations against three common pathogens (*E. coli*, *S. aureus,* and a *Bacillus* spp.) was evaluated using the disc diffusion method, and the results are presented in Table 4.

It may be noticed that both formulations presented inhibition on the growth of *S. aureus* and *Bacillus* spp. strains indicating the release of the two cannabinoid preparations into the medium but not on *E. coli*. The absence of growth inhibitory activities of cannabigerol and cannabidiol has been earlier reported in the literature [49]. Also, it was observed that CBG has a marginally stronger antibacterial effect on *S. aureus,* whereas CBD had a better effect on the *Bacillus* spp. tested. However, detailed explanations for these differences are beyond the scope of this article.

## 4. Conclusions

The therapeutic potential of CBD and CBG has been explored for different medical applications. However, limited studies have been conducted on their wound healing action. The extreme hydrophobicity of cannabinoids complicates their incorporation in aqueous topical formulations. Therefore, the concepts of nanonization and aqueous solubility enhancement by incorporating them into an amphiphilic polymer are highly promising for their efficient transdermal delivery. In this study, a novel wound dressing based on SA and loaded with cannabinoid nanoparticles was developed and studied for its possible wound healing action. 3D printing is a feasible process for producing wound dressings with flexible sizes and shapes. Also, it offers the advantage of incorporating actives such as antimicrobial, anti-inflammatory, and tissue regeneration agents that facilitate the wound healing procedure. Mono-disperse CBD and CBG nanoparticles were successfully prepared and integrated into 3D-printable inks. The nanoparticles presented enhanced thermal stability, as well as excellent release profiles, drug loading, and entrapment efficiency. Cell scratch assay indicated that in concentration 0.1 mg/mL, CBD nanoparticles do not present cytotoxicity in the first 24 h, whereas CBG nanoparticles are safe even after 48 h, indicating that CBG has a better effect on the HaCaT cell line. For both cannabinoids in the cell scratch assay, the initial wound diameter decreased in the first 6 h, then increased to levels higher than the initial ones. Short-term exposure to CBD and CBG nanoparticles seems to have a positive effect on the wound area and holds promise for future topical and emergency applications. The fabricated 3D printed films hold merit for further exploitation.

## Figures and Tables

**Table 1:** Ingredient concentrations in the loaded films.

Formulation Code	Component Concentration		
	SA (% *w*/*v*)	Calcium Chloride (% *w*/*v*)	Cannabinoid (mg/mL)
M1	6	0.4	4
M2	6	0.4	8
M3	6	0.4	12

**Table 2:** Size and PDI values of different cannabinoid suspension formulations.

**Preparation Method (Cannabinoid:PF127 1:1)**				
	**CBD**		**CBG**	
	**PdI**	**Z-Average Size (nm)**	**PdI**	**Z-Average Size (nm)**
**Method A**	0.203	166	0.202	188
**Method B**	0.272	127	0.247	195
**Cannabinoid:PF127 Ratio Comparison (Method A)**				
	**CBD**		**CBG**	
**Cannabinoid Ratio (% *w*/*v*)**	**PdI**	**Z-Average Size (nm)**	**PdI**	**Z-Average Size (nm)**
100	0.634	663	0.539	1270
90	1.000	252	1.000	845
80	0.511	410	0.649	4550
70	0.846	120	0.724	1200
60	0.554	364	0.635	4300
50	0.203	166	0.485	282
40	0.406	154	0.850	265
30	0.380	189	0.651	182
20	0.281	144	0.603	189
10	0.254	65.5	0.118	35.2
0	1.000	148	0.110	68.7
**Dilution in Water Comparison (Method B)**				
	**CBD**		**CBG**	
**Cannabinoid Ratio (% *w*/*v*)**	**PdI**	**Z-Average Size (nm)**	**PdI**	**Z-Average Size (nm)**
0.4	0.355	186	0.374	346
0.8	0.316	221	0.265	264
4	0.247	190	0.185	192

**Table 3:** Average thickness, weight, variation of the expected shape and porosity of the 3D-printed films.

	Value	STD
Thickness	0.8 mm	±0.002
Weight	0.73 g	±0.150
Shape Fidelity Assessment	7.51%	±0.150
Porosity	11.00	±0.002

**Table 4:** Growth Inhibition zones of CBG and CBD formulations on *E. coli*, *S. aureus* and *Bacillus* spp. strains.

Formulations	Growth Inhibition Zone (mm)		
	*E. coli*	*S. aureus*	*Bacillus* spp.
Control	-	-	-
CBD (5 mg/mL)	-	12.7 ± 1.2	11.7 ± 0.6
CBG (5 mg/mL)	7 ± 0.1	13.3 ± 1.5	10.3 ± 0.6

## Supplementary Materials

The following supporting information can be downloaded at: https://www.mdpi.com/article/10.3390/pharmaceutics14081637/s1, Figure S1: Variation of films’ relative weight with time in PBS presence.
