# Supplementary figures and images for: Fabrication and Preliminary In Vitro Evaluation of 3D-Printed Alginate Films with Cannabidiol (CBD) and Cannabigerol (CBG) Nanoparticles for Potential Wound-Healing Applications
Source: Pharmaceutics. 2022 Aug 5;14(8):1637. doi: 10.3390/pharmaceutics14081637 (PMC9416381; doi:10.3390/pharmaceutics14081637)

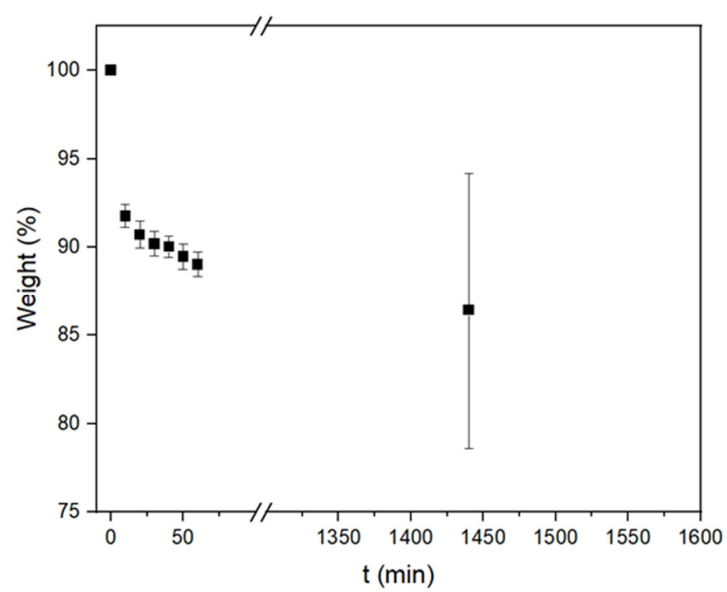

**Figure S1.** Variation of films' relative weight with time in PBS presence.

Supplement: Supplementary file 1 [file pharmaceutics-14-01637-s001.zip › pharmaceutics-1828384-supplementary.pdf]
